# Development of Severe Combined Immunodeficient (SCID) Pig Models for Translational Cancer Modeling: Future Insights on How Humanized SCID Pigs Can Improve Preclinical Cancer Research

**DOI:** 10.3389/fonc.2018.00559

**Published:** 2018-11-30

**Authors:** Adeline N. Boettcher, Crystal L. Loving, Joan E. Cunnick, Christopher K. Tuggle

**Affiliations:** ^1^Department of Animal Science, Iowa State University, Ames, IA, United States; ^2^Food Safety and Enteric Pathogens Unit, National Animal Disease Center, Agricultural Research Service, United States Department of Agriculture, Ames, IA, United States

**Keywords:** severe combined immunodeficiency, swine, humanization, cancer, xenograft, pre-clinical, animal model

## Abstract

Within the last decade there have been several severe combined immunodeficient (SCID) pig models discovered or genetically engineered. The animals have mutations in *ARTEMIS, IL2RG*, or *RAG1/2* genes, or combinations thereof, providing SCID pigs with NK cells, but deficient in T and B cells, or deficient in NK, T, and B cells for research studies. Biocontainment facilities and positive pressure isolators are developed to limit pathogen exposure and prolong the life of SCID pigs. Raising SCID pigs in such facilities allows for completion of long-term studies such as xenotransplantation of human cells. Ectopically injected human cancer cell lines develop into tumors in SCID pigs, thus providing a human-sized *in vivo* model for evaluating imaging methods to improve cancer detection and therapeutic research and development. Immunocompromised pigs have the potential to be immunologically humanized by xenotransplantation with human hematopoietic stem cells, peripheral blood leukocytes, or fetal tissue. These cells can be introduced through various routes including injection into fetal liver or the intraperitoneal (IP) space, or into piglets by intravenous, IP, and intraosseous administration. The development and maintenance of transplanted human immune cells would be initially (at least) dependent on immune signaling from swine cells. Compared to mice, swine share higher homology in immune related genes with humans. We hypothesize that the SCID pig may be able to support improved engraftment and differentiation of a wide range of human immune cells as compared to equivalent mouse models. Humanization of SCID pigs would thus provide a valuable model system for researchers to study interactions between human tumor and human immune cells. Additionally, as the SCID pig model is further developed, it may be possible to develop patient-derived xenograft models for individualized therapy and drug testing. We thus theorize that the individualized therapeutic approach would be significantly improved with a humanized SCID pig due to similarities in size, metabolism, and physiology. In all, porcine SCID models have significant potential as an excellent preclinical animal model for therapeutic testing.

## Introduction

A new field of personalized medicine has been evolving over the last decade, especially with respect to advances in individualized cancer therapies, ranging from T cell and NK cell immunotherapies, targeted monoclonal antibody therapy, and newly developed small molecule drugs. As progress is made toward the development of cancer therapies, it is critical that preclinical animal models can dependably represent human responses to drugs. Presently, mice are the most commonly used model for preclinical animal drug trials (1). However, many preclinical cancer drug trials that succeed in mice fail in humans due to vast differences in physiology, metabolic processes, and size (2, 3). The drug development process is intensive; on average, 12 years of research and $1–2 billion is required to bring a new drug to market (4, 5). To maximize the efficiency of preclinical drug and therapy testing, large animal models that better parallel human physiology are needed.

Mice with severe combined immunodeficiency (SCID) are an extremely versatile animal model for the field of cancer biology, although they pose significant limitations. The ability to engraft SCID mice with a human immune and/or cancer cell lines has made them an invaluable model for research (6, 7). Although mice are important for initial studies in different cancer fields, they are often not good models for specific aspects of human oncology (2, 8). Limitations of mouse models of cancer include small size, difficulties in modeling human tumor heterogeneity (9) and metabolic differences to humans (10, 11).

Large animal models can be more costly than murine studies, thus murine studies remain valuable for first line screens. However, testing in larger animal models is warranted to better predict outcomes in human and should be used in follow-up studies as an alternative animal model (12). Immunocompetent and SCID pigs are now being developed for human disease research purposes (13–18). Swine are more similar to humans with respect to size, anatomy, genetics, and immunology, therefore immunodeficient pigs may be a superior animal model for preclinical testing of cancer therapeutics (19–21).

Within the last decade there have been numerous SCID pig models created (16–18, 22–28) or discovered (29, 30). One of the hurdles to working with SCID pigs is maintaining viability due to susceptibility to disease. The use of positive-pressure biocontainment facilities (31) and standard animal isolators (27) have improved SCID pig health and viability. The ability to house immunodeficient pigs in a controlled environment increases their lifespan allowing them to be utilized for long-term biomedical research. Pigs are comparable in size to humans, have more similar metabolism to humans than mice (32, 33), and can be transplanted with larger human tumors.

In this review we describe the different SCID pig models that have been reported in recent years, as well as published methods established to raise SCID pigs for use in long-term research trials of 6 months or more. We describe the importance of human tumor or cancer cell xenotransplantation and how researchers can utilize immunodeficient pigs for translational studies relevant to human patients. In addition to tumor xenografts, the SCID pig has the potential to be engrafted with a human immune system, or “humanized,” just as numerous SCID mouse models have been humanized. While there is no published research on the development of a humanized SCID pig, substantial progress is being made toward this endeavor. We describe the different methods of humanization that could be used in SCID pigs, including fetal liver and intraperitoneal (IP) injections, as well as intravenous (IV), IP, and intraosseous (IO) injection in piglets. Despite the early developmental stage for humanized SCID pigs, the SCID pig has vast potential to be utilized for translational oncology. Our overarching hypothesis in this review is that porcine SCID models will be more translational than mouse models for oncology research in the future.

## Existing SCID pig models

### Previously described and generated SCID pigs

**Figure 1:**
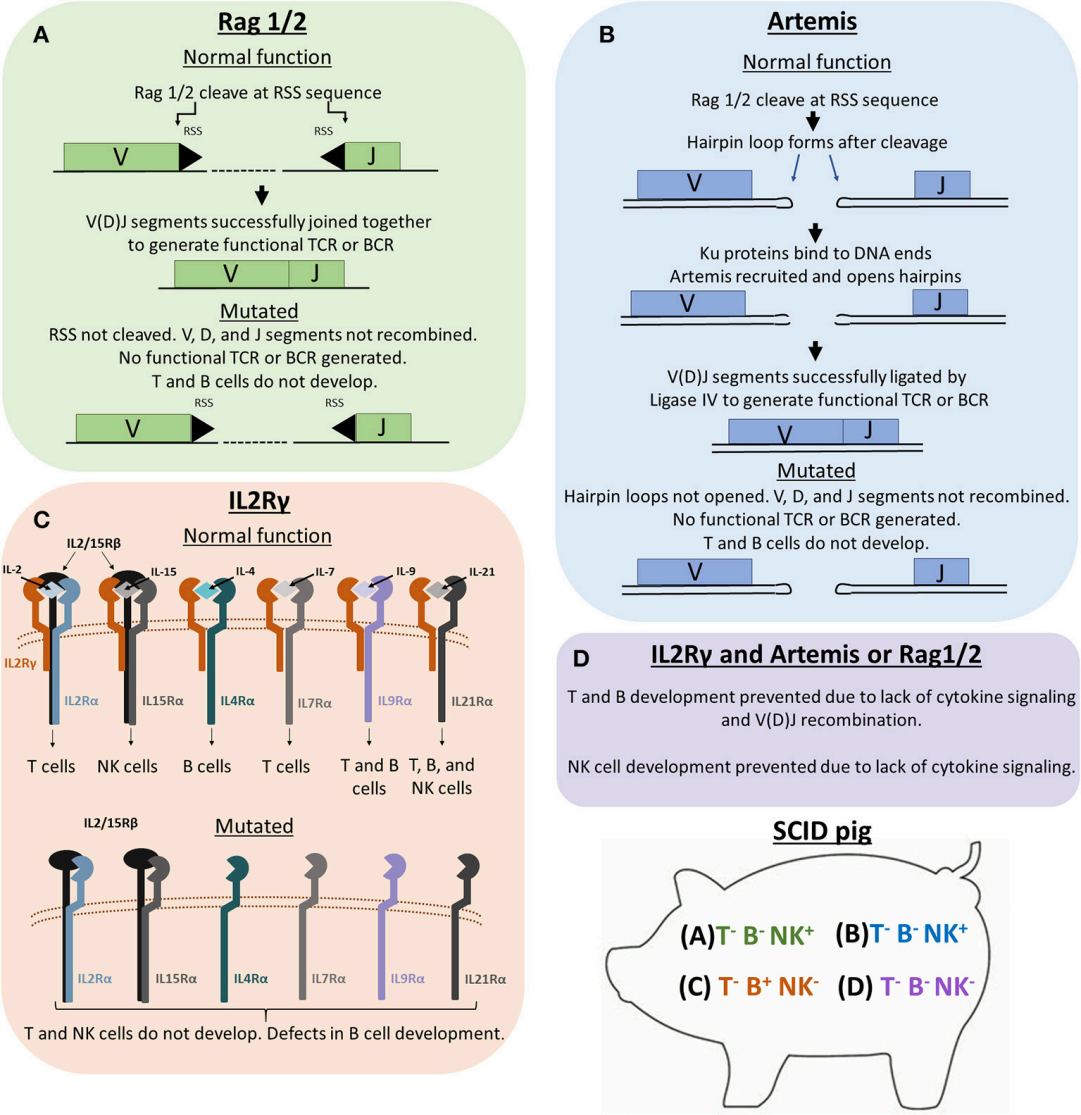
Genetic and molecular mechanisms of Rag1/2, Artemis, and IL2Rγ in lymphoid development. Previous SCID pig models have been generated or described with mutations in *ARTEMIS, RAG1, RAG2*, or *IL2RG*. **(A)** Rag1 and 2 are subunits of an endonuclease that cleave recombination signal sequences (RSS) flanking V, D, and J gene segments. Cleavage of RSS sequences are required for the gene segments to be joined together. Non-functional Rag1 or 2 proteins cannot cleave these sequences, therefore preventing T cell receptors (TCRs) and B cell receptors (BCRs) from forming. T cells and B cells cannot develop due to non-functional TCR and BCR rearrangement. **(B)** Artemis is an endonuclease that is responsible for the cleavage of hairpin loops that form after Rag1 and 2 cleaves RSS sequences. These hairpin loops must be cleaved in order for Ligase IV to ligate V, D, and J gene segments together. If Artemis is not functional, these hairpin loops cannot be cleaved, which prevents TCR and BCR rearrangement. **(C)** IL2Rγ is a subunit required in the receptors for IL-2, IL-15, IL-4, IL-7, IL-9, and IL-21. Without functional IL2Rγ, developing cells that require these cytokines for development (mainly T, B, and NK cells) are not receptive to cytokine signaling, which prevents proper differentiation of T, B, and NK cells. **(D)** Pigs with mutations in both a VDJ recombination gene (*RAG1/2* or *ARTEMIS*) and *IL2RG* lack T, B, and NK cells.

**Figure 2:**
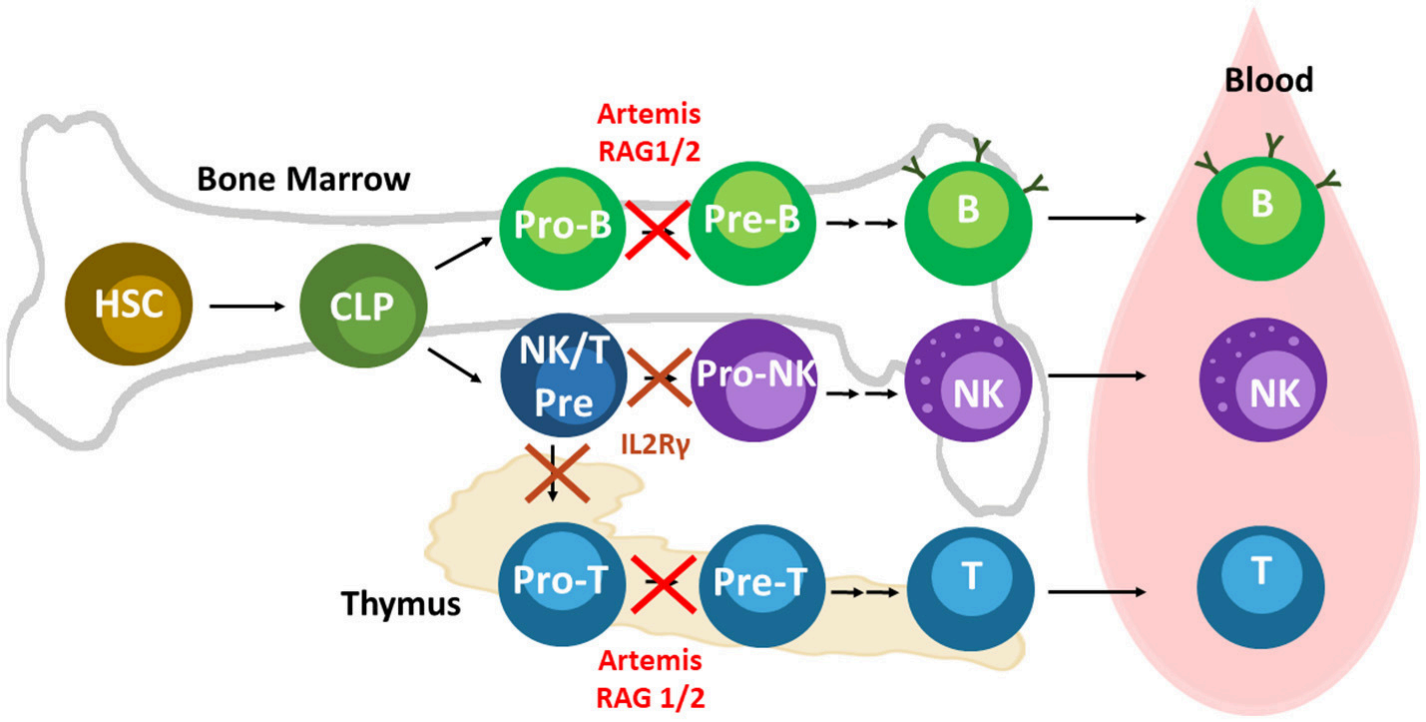
Lymphoid development and relevant SCID pig mutations. Mutations in Artemis, RAG1/2, and IL2Rγ leads to SCID in pigs. Artemis and Rag1/2 are active in Pro-B and -T cells during differentiation. IL2Rγ is required at an earlier stage of development than RAG1/2 and Artemis. NK cells and T cells both require cytokine signaling through IL2Rγ early in differentiation. Mutations in IL2Rγ prevent differentiation of T and B cells. Mouse B cells appear to rely on IL2Rγ signaling more than human and pig B cells. B cells can still develop in humans and pigs with mutations in IL2Rγ, although they are mostly non-functional due to the absence of helper T cells.

Within the last decade, numerous SCID pig models have been developed through mutagenesis or discovery of natural mutations. These SCID pig models are outlined in Table 1. Figure 1 shows the genetic and molecular mechanisms for the mutations described below that cause SCID, and Figure 2 shows the differentiation step blocked by each of these mutations.

**Table 1:** Previously described SCID pig models.

**Mutation(s)**	**Mutagenesis method**	**Cellular phenotype**	**Breed**	**Rearing method**	**Oldest age reported**	**References**
*IL2RG*	Gene targeting vector	T – B+ NK–	Landrace × Large White	Conventional housing	54 days	(16)
*ARTEMIS*	Natural	T – B – NK+	Yorkshire	Biocontainment bubble	6 months	(29, 30)
*IL2RG*	Zinc finger nuclease	T – B+ NK–	Cross bred	Did not rear	neonatal	(18)
*RAG 1/2*	TALENs	T – B–	Bama miniature	Conventional housing	29 days	(22)
*RAG 1*	Gene targeting vector	T – B–	Duroc	Did not rear	neonatal	(23)
*RAG 2*	TALENs	T – B– NK+	Minnesota minipig	Conventional housing	29 days	(24)
*IL2RG*	CRISPR/Cas9	T – B+ NK–	Cross bred	Conventional housing	12 days	(17)
*RAG 2*	Gene targeting vector	T – B – NK+	Cross bred	Conventional Housing	12 weeks	(25)
*RAG 2 IL2RG*	CRISPR/Cas9	T – B – NK−	Yorkshire cross breed	Gn Isolator	34 days	(26)
*IL2RG*	Zinc finger nuclease	T – B+ NK−	Cross bred	Isolator	103 days	(18, 27)
*ARTEMIS IL2RG*	Natural and CRISPR/Cas9	T – B – NK−	Yorkshire	Biocontainment bubble	18 days	(28)

The first SCID pig was described in 2012 (13) after a serendipitous discovery in an infection study (29). To confirm the lack of a functional immune system, these SCID pigs were transplanted with human cancer cell lines. Injected cells were not rejected and developed into tumors in the SCID pigs (13). After further analysis, it was found that the discovered SCID pigs had two naturally occurring mutations in two separate alleles within the *Artemis (DCLRE1C)* gene, which leads to SCID either in the homozygous or compound heterozygous state (30).

Artemis is required for DNA repair during T and B cell development. Specifically, during the process of VDJ recombination, after RAG1/2 nucleases cleave DNA at the RSS sequences flanking V, J (and sometimes D) segments (34), a hairpin loop then forms at the end of the double stranded break (DSB). Ku70/80 proteins are recruited to the area of the DSB along with Artemis protein, which is responsible for cleaving the hairpin loop so it can be ligated by Ligase IV (35). Without functional Artemis, these hairpins are not cleaved, and functional V, D, and J joins cannot be made. Lack of Artemis function leads to a cellular profile in which T and B cells are deficient, but NK cells develop (T^−^ B^−^ NK^+^) and are functional (29, 30, 36). Homozygous or compound heterozygous *Artemis* pigs can be raised to 6 months of age in biocontainment facilities developed at Iowa State University [31, unpublished observation].

Another SCID pig was also described in 2012 with an engineered mutation within the *IL2RG* gene (16). In humans and mice, the IL2 receptor γ (IL2Rγ) subunit is required for IL-2, IL-4, IL-7, IL-9, IL-15, and IL-21 signaling (37). The *IL2RG* gene is on the X chromosome in mammals and the receptor is expressed on lymphoid cells, including developing cells. The cytokines noted are required for proper lymphoid development, and thus deletion of the IL2Rγ subunit disrupts development of T and NK cells, and B cells to a variable extent (38, 39). The cellular phenotype of these *IL2RG* knockout pigs was T^−^ B^+^ NK^−^, similar to humans (38, 39). B cells in *IL2RG* knockout SCID pigs were not able to secrete immunoglobulin nor class switch due to absence of helper T cells (16). Interestingly, cloned heterozygous *IL2RG*^+/−^ females exhibited SCID-like phenotypes, which was attributed to aberrant X-inactivation. These females were raised to sexual maturity and crossed with WT males; female *IL2RG*^+/−^ offspring from this cross phenotypically resembled WT animals (16). This finding emphasizes the importance of monitoring for SCID phenotype status in cloned piglets even if the expected outcome is a carrier animal. Other groups have also introduced mutations in the *IL2RG* gene by CRISPR/Cas9 (17) and zinc finger nuclease (18) methods, and the resulting pigs also displayed cellular phenotypes of T^−^B^+^NK^−^. Animals in these studies were raised in conventional settings and had lifespans that ranged from 12 days to 7 weeks (16–18).

The recombination activating genes, *RAG1* and *RAG2*, have previously been mutated to create pig models. They code for subunits of a nuclease (RAG1/2), that is involved in VDJ recombination required for T and B cell receptor (TCR and BCR, respectively) generation (40). Without functional RAG1/2 nuclease, VDJ recombination does not initiate, and T and B cells do not develop (41, 42). Homozygous or biallelic *RAG1* or *RAG2* SCID pigs lacked IgM^+^ B cells and CD3^+^ cells in peripheral blood (22, 23, 25). NK cells were present in these animals and were classified as either CD3^−^ CD8α^+^ (22) or CD16^+^ CD8α^+^ (25). RAG knockout pigs were generated with either TALENs (22, 24), gene targeting vectors (25), or CRISPR/Cas9 (26) mutagenesis methods. Previous *RAG1* or *RAG2* mutant SCID pigs have been raised to 29 days (22, 24) to 12 weeks (25) of age in conventional housing.

Once single mutant pigs were established, research groups began to introduce mutations in both VDJ recombination pathway genes (*RAG1/2* or *ARTEMIS*) and *IL2RG* to produce pigs that lacked innate and adaptive immune function, generating T^−^ B^−^ NK^−/lo^ SCID pigs (26, 28). Double-mutant pigs are an important animal model to develop, as rodent models of SCID mice lacking NK cells, as well as T and B cells, engraft human cells better than T^−^B^−^ NK^+^ models (43). It is therefore of interest to generate a T^−^ B^−^ NK^−^ SCID pig model for humanization studies. In 2016, *RAG2/IL2RG* knock out piglets were generated and used in pathogenesis study with human norovirus (26). *RAG2/IL2RG* SCID pigs lacked T and B cells, and there were decreased numbers of NK cells compared to controls. The presence of some NK cells was attributed to a hypomorphic mutation within IL2Rγ (26). Our group has recently engineered a complete *IL2RG* knockout that was introduced into an *ARTEMIS* null genetic background resulting in SCID pigs that lack T, B, and NK cells (28).

### Methods for SCID pig rearing

**Figure 3:**
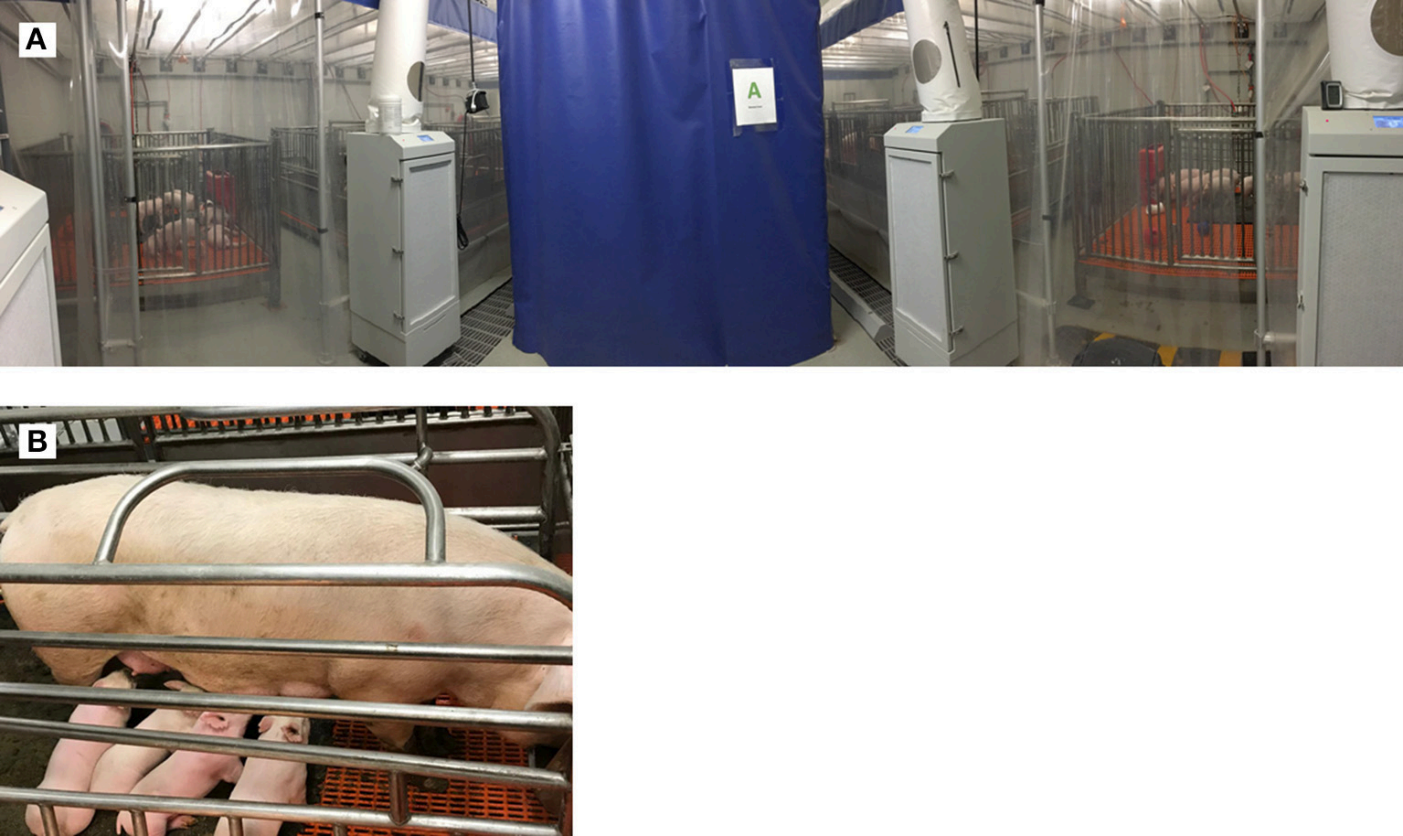
Biocontainment facilities for rearing SCID pigs. **(A)** Biocontainment facilities for the rearing of *ARTEMIS*^−/−^ SCID pigs. **(B)** SPF female *ARTEMIS*^+/−^ carriers nursing 3 week old SCID and non-SCID piglets after naturally farrowing in biocontainment facilities.

One of the difficulties to overcome when using SCID pigs in research is maintaining animal viability. SCID pigs raised in conventional settings typically succumb to disease between 6 and 12 weeks of age [unpublished observation, 17]. Biocontainment facilities have been specifically designed to limit exposure of Iowa State University's *ARTEMIS*^−/−^ SCID pigs to any micro-organisms (Figure 3A). These rooms have positive-pressure HEPA filtered air flow into a containment bubble and all water entering the bubble is UV irradiated and filtered through a 0.5 μm filter. Personnel entering the bubble wear appropriate garments to limit introduction of organisms into the room, including room dedicated protective suits, hair net, surgical mask, gloves and rubber boots (31). Piglets are derived either by snatch farrowing (caught in a sterile towel as they are delivered vaginally) or by cesarean section and are transferred immediately into a sterilized bubble. Piglets are immediately fed pasteurized colostrum for the transfer of maternal immunoglobulin (44), fed sterile milk replacer for 21 days, and then transitioned to irradiated feed, which is continued throughout life (31). Specific pathogen-free (SPF) *ARTEMIS*^+/−^ carrier females have been raised to sexual maturity and are able to naturally farrow *ARTEMIS*^−/−^ SCID litters within the ISU bubble facilities (Figure 3B). *ARTEMIS* mutant SCID pigs can be successfully reared to 6 months of age in these facilities (unpublished observation).

Survivability of previous *IL2RG* knock out pigs has varied from 2 to 7 weeks (16) and derivation of animals and available housing likely impacts outcome. Recently Hara et al. (27) used small isolators and developed piglet delivery protocols to help extend the lifespan of *IL2RG* knock out SCID pigs. To achieve this goal, excised uteruses were brought into isolators units, piglets were delivered, and reared within these isolators. One SCID piglet raised in the isolators was raised to a planned endpoint of 12 weeks of age without incidence of bacterial or fungal disease (27).

## SCID pig cancer xenotransplantation studies

### Existing immortal cell lines develop into tumors in SCID pigs

Since the generation of SCID pigs is so recent, there are only a few studies that have been published on the ability of SCID pigs to accept human xenografts. The first SCID pig xenograft study involved the transplantation of human melanoma (A375SM) and pancreatic carcinoma cell (PANC-1) into the ear tissue of *ARTEMIS*^−/−^ SCID pigs (13). All SCID pigs receiving cancer cells developed tumors at the site of injection, thus establishing an orthotopic model of melanoma that could be studied further (13). Additionally, the ability of ovarian carcinoma cell line OSPC-ARK1 to develop tumors in *ARTEMIS*^−/−^ SCID pigs was explored. SCID pigs were injected in the ear and neck muscles with OSPC-ARK1 cells and subsequently monitored for tumor development. In 3 of the 4 SCID pigs injected, tumors developed within 30 days, with a shortest time of 7 days to palpable tumors. Biopsy samples revealed the ovarian tumors in SCID pigs expressed diagnostic markers commonly used in human cancer diagnoses, and tumors in SCID pigs resembled human tumors (45).

Pigs biallelic for *RAG2* mutations can engraft human induced pluripotent stem cells (iPSCs). Injected iPSCs developed teratomas that represented endoderm, mesoderm and ectoderm tissues (24). Teratomas were grossly visible 12 days after cell inoculation for one recipient; and about 7.5 weeks in the other recipient. Histological analysis revealed CD34^+^ and CD45^+^ cells developed in the teratoma, (24), indicating that human immune lineage can survive and differentiate in *RAG2* knockout pigs. This important finding indicates that SCID pigs can accept various types of human xenografts. In a follow-up study, *PERFORIN*, and *RAG2* double knock out (*Pfp/RAG2* dKO) mice and *RAG2* knock out pigs were compared for their ability to engraft human iPSCs. The *RAG2*^−/−^ pigs developed teratomas from injected iPSCs at a higher rate than the *Pfp/RAG2* dKO mice. Human teratomas that developed in the *RAG2* knockout SCID pigs also had a higher prevalence of CD45^+^ and CD34^+^ cells in the teratoma than in SCID mice (46). Thus, the *in vivo* environment in pigs supports the growth and differentiation of human cells, and in some instances, is an improved system over SCID mice.

## Porcine immunological similarities to human

**Figure 4:**
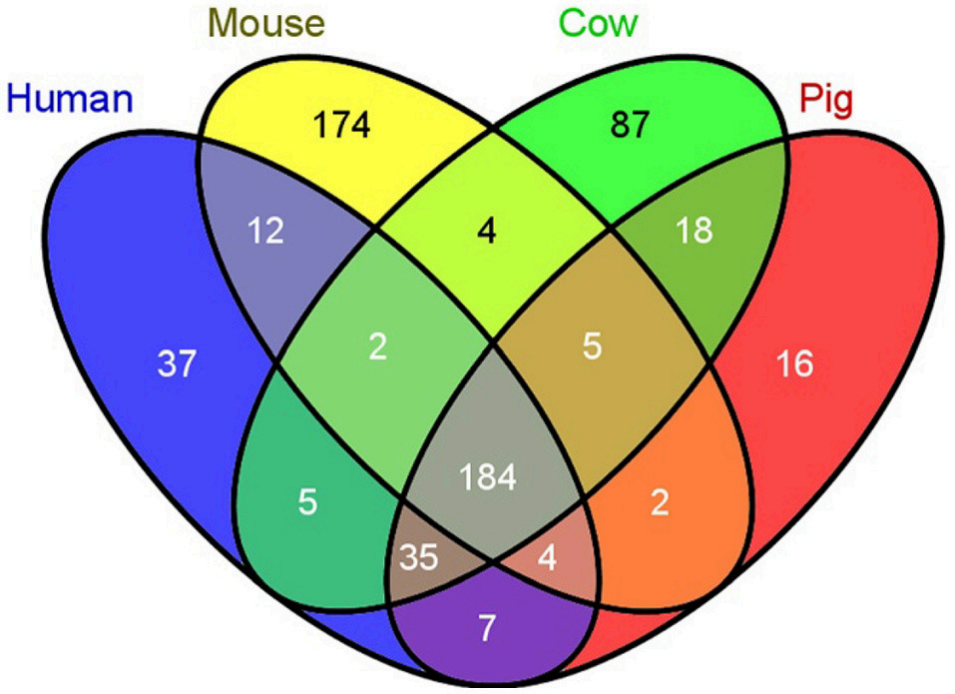
Swine have fewer unique immunological genes compared to humans than do mice. A comparison of the number of unique immunological genes was compared between humans, mice, cows, and pigs. Pigs have 2 times less unique genes, while mice have 4.7 times more unique genes compared to humans [Reprinted from Dawson et al. (20); Figure 1).

Several aspects of the pig immune system are more similar to humans than mice, providing another advantage of swine models for research (39). Humans and pigs have higher sequence orthology for immune-related genes (termed the “immunome”) than humans and mice (20). Immunome-specific gene family expansions, a measure of evolutionary divergence, have occurred in pig relative to human at half the rate detected in mouse or cow (20), and pigs have significantly fewer unique genes not found in humans when compared to unique gene abundance in cow or mouse (Figure 4). Additional analyses have further expanded human and pig similarities, although absence of two inflammasome gene families have also been found uniquely in the pig genome (47). As well as immunome structural similarities, immune responses are highly comparable between human and pig [reviewed in (41)]. For example, the transcriptomic response to lipopolysaccharide of pig macrophages *in vitro* is more similar to human responses as compared to mice. Specifically, clusters of genes with IDO1 as hub were detected in human and pig macrophage responses, but not in mice, while a NOS2A-related gene cluster was only found in the mouse macrophage LPS response (48).

**Figure 5:**
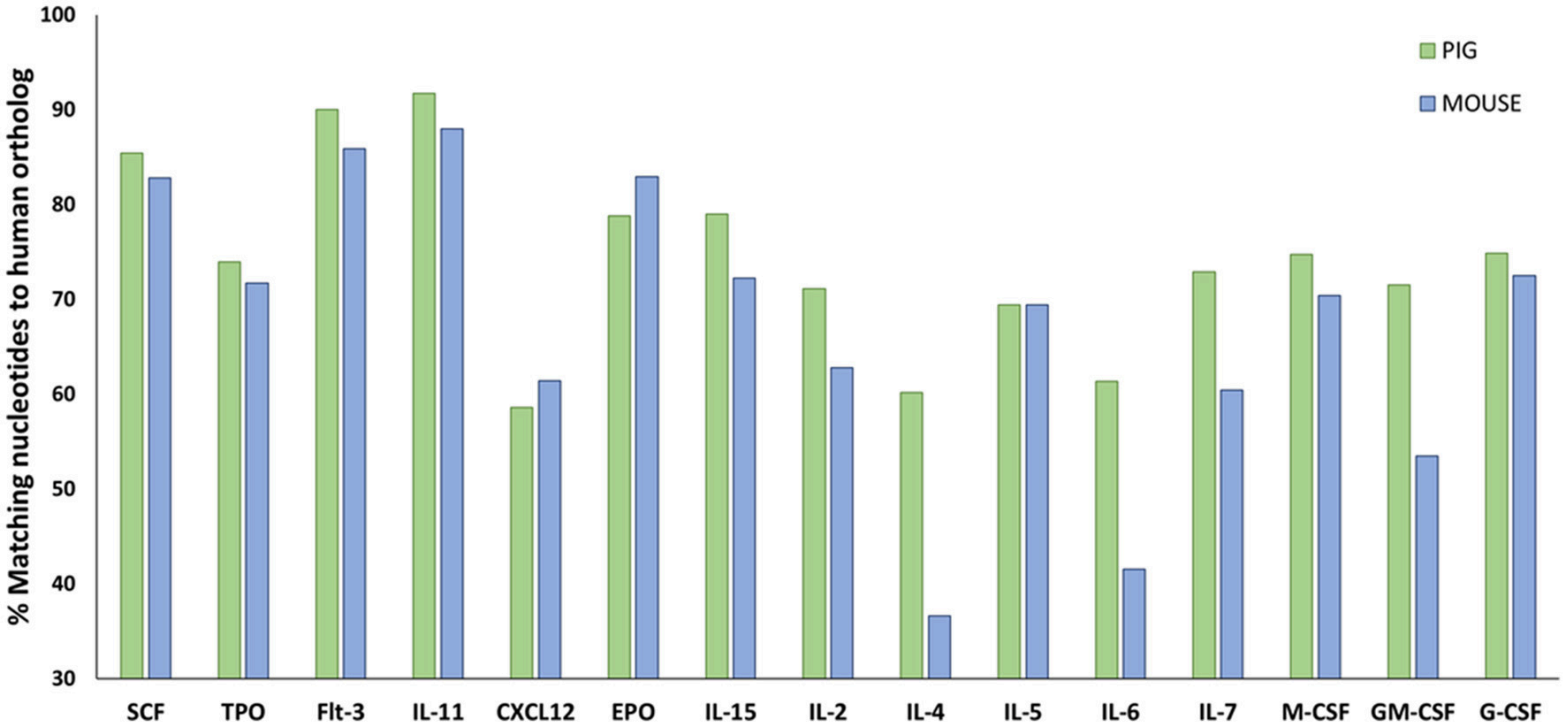
Amino acid sequence comparisons in hematopoietic cytokines for pig and mouse compared to humans. Amino acid sequences for relevant hematopoietic cytokines and other ligands were acquired from Ensembl (https://useast.ensembl.org/index.html). The percentage of matching sequence between humans to pigs and mouse is shown above. Porcine shares higher sequence similarity to humans for a majority of hematopoietic cytokines compared to mice. Supplemental Table 1 shows the accession numbers from the sequences that were compared.

Human hematopoietic stem cell (HSC) development in swine for humanizing pigs will be dependent on swine cytokine signaling. Hence, it is important to determine the cross reactivity of porcine cytokines with human cells. Protein sequence analysis shows that swine share more homology in cytokines involved in hematopoiesis with humans than mice (Figure 5; Supplemental Table 1), which suggests that certain human lineages may differentiate with greater success in SCID pigs than in SCID mouse models.

## Routes for humanization and applications

Given the high similarity of swine and human immune genes, we would anticipate that human HSCs transferred into SCID pigs would successfully engraft and differentiate into representative human immune cell types. Current building of swine SCID models relies heavily on translating methods used for mouse humanization to generate new humanized SCID pig models. To humanize the mouse, three different approaches are utilized (6, 7). These methods include transfer of purified human CD34^+^ stem cells, peripheral blood leukocytes (PBLs), or transfer of fetal bone marrow, liver, spleen, and lymph node tissues. Just as in SCID mouse models, these same approaches and cell types can be investigated as methods to humanize SCID pigs. The pig immune signaling molecules that support engraftment are expected to be similar to humans, thus we expect successful development of human immune cells.

Currently the NOD-SCID-IL2Rγ (NSG) knockout mouse is the gold standard model for humanization. The *Sirpa* allele in the NOD background contains polymorphisms that allow the encoded Sirpa protein to bind to human CD47, which then sends a inhibitory signal that prevents phagocytosis of human cells (49, 50). Swine SIRPA also binds to human CD47 (51), so we speculate that porcine SIRPA-dependent phagocytosis of human cells would not be a barrier to SCID pig humanization.

**Figure 6:**
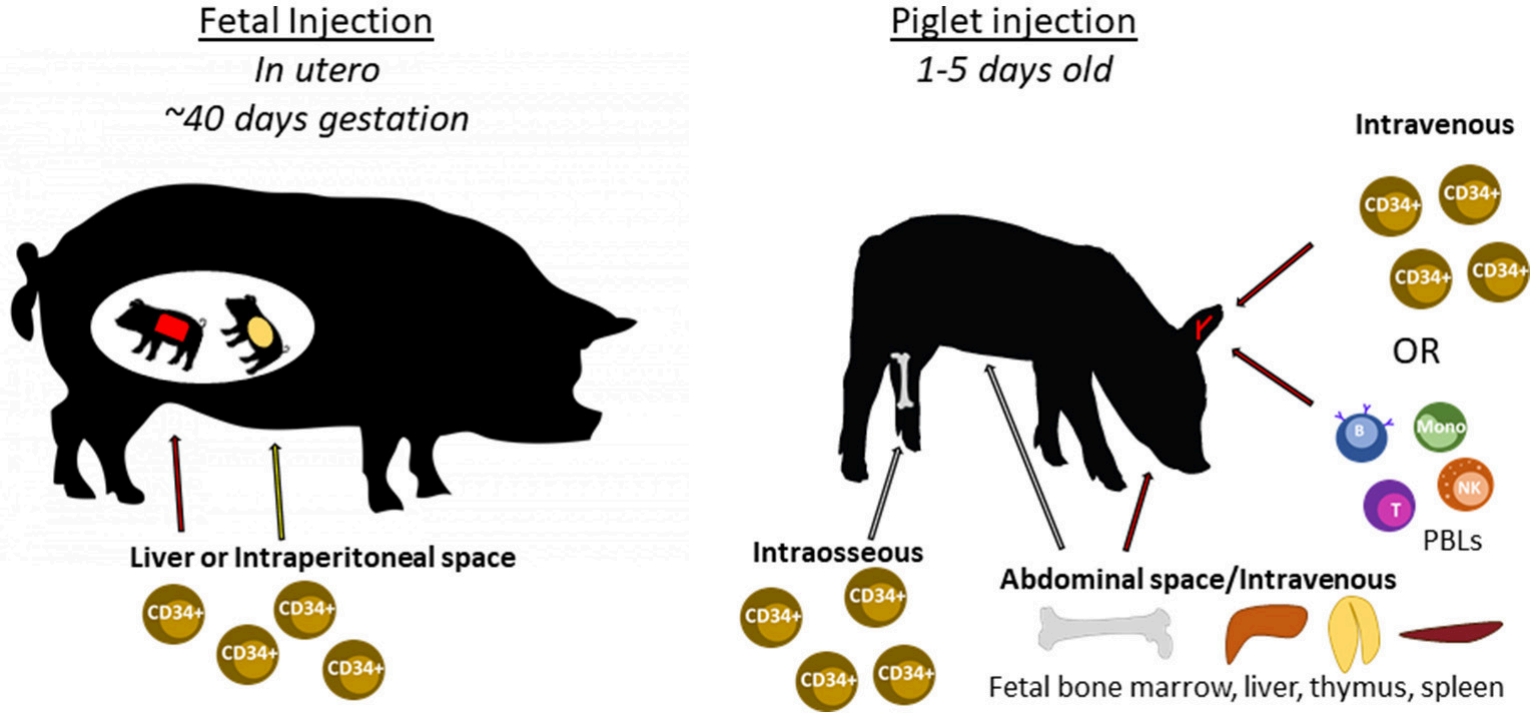
Cell types and routes of injection for SCID pig immunological humanization. Swine can be injected with human cells at two different developmental stages. During gestation, fetal piglets at ~40 days of gestation can be injected with human CD34^+^ stem cells within either the liver or intraperitoneal space via ultrasound guidance. Newborn piglets can also be injected with human CD34^+^ stem cells through either intravenous or intraosseous routes. PBLs can also be injected via intravenous injection. Fetal tissues including bone marrow, liver, thymus, or spleen can be transplanted within the abdomen, potentially under the kidney capsule as is done with SCID mice.

The following sections describe previous humanization methods performed in SCID mice and other large animal models, and how these methods can be utilized to humanize SCID pigs. Figure 6 shows an overview of different human immune cell types and anatomical injection sites for SCID pig humanization. Past studies utilizing injection of human HSCs or human induced pluripotent stem cells into large animal models are presented in Table 2.

**Table 2:** Previously described human stem cell injection studies in swine and sheep.

**Large animal**	**Type of human cells injected**	**Number of cells injected**	**Age and injection site**	**Human cell type(s) that differentated**	**References**
PI sheep	Fetal liver cells	0.2–1 × 10^10^/kg	Fetal peritoneal cavity	T, B, NK, myeloid, erythroid	(52)
PI pig	CD34^+^ cells from cord	0.5–3 × 10^6^	Fetal intraperitoneal cephalad space	T, B, NK, Myeloid	(53)
PI pig	T cell depleted bone marrow	5 × 10^7^ (5 × 10^9^/kg)	Fetal peritoneal cavity	B	(54)
PI sheep	CD34^+^ cells from bone marrow	3.6 × 10^6^	Fetal peritoneal cavity	T, B, myeloid,dendritic cells, erythroid	(55)
PI pig	T cell depleted bone marrow or cord blood	5 × 10^7^ (5 × 10^9^/kg)	Fetal peritoneal cavity	T, B, NK, myeloid	(56)
PI sheep	ESC-derived CD34^+^	3 × 10^6^	Fetal peritoneal cavity	T, B, NK, Monocytes, neutrophils	(57)
SCID pig	iPSCs	5–10 × 10^6^	Piglet ear and lateral flank	CD34^+^, CD45^+^	(24)
SCID pig	iPSCs	10 × 10^6^	Piglet ear and lateral flank	CD34^+^, CD45^+^	(46)

*PI, Pre-immunocompetent; ESC, embyronic stem cells; iPSC, induced pluripotent stem cells*.

### CD34^+^ cell injection via fetal liver and intraperitoneal space

Successful humanization of SCID pigs will require that human HSC be injected into sites of hematopoiesis in the pig. During gestation the initial location of hematopoiesis is the yolk sac (58). As gestation continues, the fetal liver becomes the site of hematopoiesis, typically around the beginning of the second trimester (59–63). During swine gestation, hematopoiesis begins at day 30 in the fetal liver (62). Intrauterine injection of human hematopoietic cells during the fetal liver phase of hematopoiesis would provide a rich environment for human stem cells to engraft and differentiate (64), as supporting cells in the fetal liver niche express c-Kit, CD34, CXCL12, and NOTCH (59). Additionally cell subsets in the fetal liver can promote hematopoiesis, such as CD34^lo^ CD133^lo^ cells that have been described in human (65). Differentiated human cells that develop in the SCID pig liver may also migrate to the bone marrow around the same time as other developing swine immune cells, which may increase the ability of human immune progenitors to engraft within the SCID pig bone marrow. Fewer human cells would be required for the fetal liver injection strategy when compared to the number of cells required to engraft a fully developed piglet. Taken together, we hypothesize that fetal injection of human hematopoietic stem cells will likely lead to the highest levels of engraftment compared to other methods described in later sections.

The first study involving *in utero* injection of human cells into a large animal was performed by Zanjani et al. (52). Human fetal liver cells were injected into the IP space of fetal sheep at days 48–54 gestation (145 day term) through the uterine wall. The recipient sheep were immunocompetent, but pre-immune at this stage of development. Two of the derived sheep were raised to 15 months of age, and human CD3^+^, CD16^+^, and CD20^+^ cells were still in circulation, albeit at very low frequencies (52). Other studies involving the transplantation of human CD34^+^ cells in the fetal liver of pre-immune sheep have resulted in similarly low levels of human cell engraftment and differentiation (55, 57).

In addition to sheep, *in utero* injection of human CD34+ cells have been performed in pre-immunocompetent conventional pigs. The first was described in 2003 (53) with the injection of cord blood derived CD34^+^ cells into the IP space of pre-immune fetal piglets at ~40 days of gestation (114 day term). Populations of human CD3^+^ cells were detected in the thymus, CD19^+^ cells and myeloid cells also developed *de novo* in the pig, in as short as 40 days post-injection. Additionally, human CD34^+^ CD45^+^ cells were isolated from pig bone marrow 120 days after transplantation and were subsequently transplanted into SCID mice with successful engraftment of human cells observed. This result indicates that the pig bone marrow environment is able to support the development of functional human HSCs (53).

Humanization of pigs could serve as a source of human T cells for immunotherapeutic use. Ogle et al. (56) depleted CD3^+^ cells from human bone marrow or cord blood and injected into the IP space of fetal piglets at 40–43 days of gestation. Human T, B, macrophages, and NK cells were detected in peripheral blood of piglets using RT-PCR by amplification of CD3, CD19, CD14, and CD16/CD56, respectively. In order to determine if the human T cells had developed *de novo*, blood was analyzed for the presence of human T cell receptor excision circles (TREC). Human TRECs were observed at a level that suggested new human T cells had developed in the swine thymus (56). Similar, studies were performed in which fetal swine were injected with human T cell depleted bone marrow (54) or T cell depleted cord blood (66), in which human cell engraftment was observed. In all, these studies show that human T cells can develop *de novo* when human HSC are injected into fetal swine.

**Figure 7:**
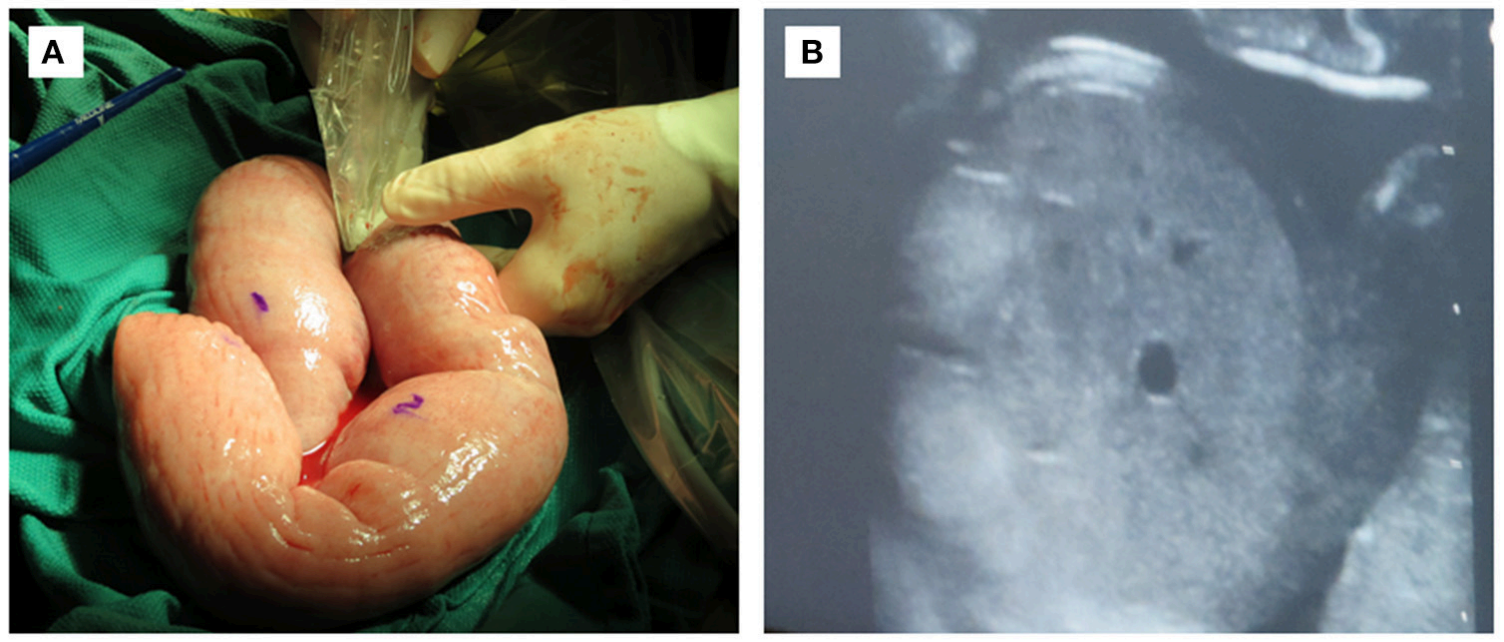
*In utero* injection of fetal intraperitoneal space via laparotomy. **(A)** Exteriorized uterus at 40 days gestation being ultra-sounded for fetuses. Water soluble marker can be used for marking fetuses. **(B)** Ultrasound image of fetal liver with which human CD34^+^ stem cells would be injected.

Successful engraftment of SCID pigs utilizing *in utero* injections requires consideration of timing and surgical procedures. We hypothesize that a humanized SCID pig could be developed via *in utero* injection of human CD34+ cells within the fetal liver or IP space at ~40 days of gestation. We have described detailed laparotomy protocols that can be followed for procedures involving stem cell injection into fetal IP space and livers [(67); Figure 7). The level of human cell hematopoiesis in a SCID pig model has yet to be determined, however it is expected that engraftment would be comparable to that described for immunocompetent animals. Given the lack of pig immune cell development in pigs with SCID, the available niches for human progenitor cells to develop in the bone marrow and thymus would be increased.

### Peripheral blood leukocyte injection via intravenous or intraperitoneal routes

In 1988, the first humanized mouse models were generated in efforts to investigate the AIDS virus interaction with its human host. One of these models described the injection of human PBLs into the IP space of SCID mice (68). Mice were injected by the IP or IV routes with 10–90 million human PBLs (termed hu-PBL SCID mice). IV injection was deemed ineffective in mice, likely due to the difficulty of proper IV administration in a mouse. Human cells injected IP in mice were able to migrate to the spleen, lymph nodes, and were also detected in peripheral blood; 4 weeks post IP injection very few human PBL were detected in the peritoneal space. Mice were vaccinated with tetanus toxoid, which PBL donors were known to be immune. Eight of 10 animals injected with PBLs produced human immunoglobulin against tetanus toxoid which supported that human helper T cells and B cells were functional in the hu-PBL SCID mice. Human CD14^+^ monocytes were also present in the spleens of mice 8 weeks post transplantation (68). These hu-PBL SCID mice are utilized in a variety of different fields including HIV (69–71), cancer (72, 73), basic immunology (74, 75), and atopic dermatitis (76).

Hu-PBL-SCID pigs could be generated by IV or IP injection of human PBLs into SCID pigs. IV injection of human cells have been deemed ineffective for engraftment in mice. However, tail veins are typically used in mice, which are difficult to properly inject. Piglets have large and visible ear, cephalic, and saphenous veins that are easily accessible. A limitation of human PBL injections in pigs could be the amount of cells required relative to the number of cells injected into mice. Mice are typically 20 g (0.02 kg), while a typical newborn piglet weighs about 1–2 kg. In previous studies, the minimum amount of human PBLs injected into mice is about 10 million cells, which scales up to 0.5–1 billion in a piglet. However, there are strategies to overcome the cell number limitation. One source for human PBLs could be leukoreduction system chambers (LRSCs), which are utilized by blood banks to remove PBLs during plateletpheresis. During a normal collection of platelets from a donor, ~2 billion PBLs can be obtained from LRSCs (77). Another approach is matching a human with a SCID pig and performing repeat PBL injections from the same human donor. Also, it is possible that the number of human PBLs required for successful engraftment of SCID pigs would not be as high as calculated from murine studies. Given that methods to obtain large numbers of PBLs are available, the number limitation is not expected to prevent development of a hu-PBL-SCID pig.

One consideration for using a SCID pig injected with human PBLs is that these animals will eventually develop graft vs. host disease (GVHD). SCID mice injected with human PBLs develop GVHD ~3–11 weeks after injection (78) while it takes 14–16 weeks in SCID rats (79). It is currently unknown how long it would take SCID pigs to develop GVHD after human PBL cell transplantation, as well as how the cellular dose would impact the GVHD time frame. This is a question that will need to be addressed as this model is developed. Another important question that will need to be addressed in developing this model is the time period required for human PBL engraftment within the SCID pig.

One benefit of the PBL model is that it could be used for short term studies in SCID pigs. SCID pigs raised in conventional settings can typically survive to 6 weeks of age. If piglets are injected with human PBLs shortly after birth (1–5 days), this would give researchers ~a 6 week window to perform experiments. It may also be appropriate to administer immunosuppressive drugs during this period of time to reduce the effects of GVHD.

### CD34^+^ cell injection via intraosseous or intravenous routes

Another route for humanization is through the injection of purified human CD34^+^ HSCs into live-born piglets. We have previously performed bone marrow transplantations (BMT) on our SCID pigs through IV injection of unfractionated pig bone marrow cells (80). One hypothesis is that human HSC could be administered in the same way to generate a humanized SCID pig. Typically in pig to pig bone marrow transplants, it takes ~10 weeks to observe a moderate increase in the number of circulating porcine lymphocytes (80). We hypothesize that human engraftment and *de novo* development of human cells would require at least 10 weeks to observe human cells in circulation based on pig to pig BMT observations. It may be of value to compare cell dosages and engraftment rates of human and pig HSC in SCID pigs. IV injection of human HSC is much less invasive than fetal injections, however it may take longer to achieve engraftment and differentiation of human cells.

Another method of human HSC administration is through intraosseous (IO) injection. IO injection of stem cells and mesenchymal stem cells have previously been performed in SCID mice (81), dogs (82, 83), and pigs (84). IO injection is also a method for bone marrow transplantation in humans (85). It is hypothesized that IO injections are preferable over IV injections due to stem cell trapping in pulmonary tissue, which is often observed in IV injections (86, 87). In addition, IO administration introduces cells to the site within which they would differentiate. Protocols have also been developed for the delivery of various substances though IO injection in swine (84, 88, 89). IO injection of human CD34^+^ cells into SCID pigs is therefore another potential route for studying engraftment and humanization models.

### Implantation of human fetal bone marrow, thymus, and liver tissues

Another potential method for humanization of SCID pigs is through the transplantation of human fetal liver, thymus, lymph node, and spleen tissue, as has been previously performed in mice (90). Such human lymphoid tissues can be transplanted into mice either by implantation under the kidney capsule or IV injection of a cellular suspension. Mice transplanted with human lymphoid tissues appear to have immunological protection, as the lifespan of transplanted mice can be extended to 17 months, compared to 4 months for non-transplanted mice. Mice injected with both human thymic and fetal liver cells developed human T and IgG secreting B cells (90). The chimeric mice with human bone marrow, liver and thymus (BLT) are used to study interactions between human immune cells and patient derived melanomas (91).

*De novo* development of human T cells within the pig requires that human T cells can differentiate within the swine thymus. Transplantation studies show that the porcine thymus supports human T cell development, as mature human T cells develop in athymic mice transplanted with porcine thymus and human HSCs (92, 93). Human T cell development within the swine thymus is particularly important for long term studies because this would allow newly differentiated human T cells to develop tolerance to pig antigens. Human thymic tissue could also be transplanted into SCID pigs for human HLA restricted T cell development. Development of GVHD is observed in mice humanized with fetal bone marrow, liver, and thymic tissue (94), potentially due to human thymus dependent T cell development. Depending on the experimental question being addressed, transplantation of a human thymus may be a preferred method in humanizing SCID pigs.

One issue with generating BLT humanization models is the limited fetal tissue availability, as well as ethical implications. Smith et al. described a way to circumvent these issues by propagating and expanding BLT tissues in one mouse and then transplanting into 4–5 other mice (95). SCID pigs could be useful in this regard as human tissues would have the potential to grow to a large enough size that they could be transplanted again into a second set of animals.

## Future outlook on the utilization of SCID pigs for cancer therapies and research

### Humanized SCID pigs for CAR-T and CAR-NK cell therapy research

Chimeric antigen receptor (CAR) T and NK cells have been developed in recent years as a cancer immunotherapy. CAR-T cells targeted against CD19 for patients with B cell lymphomas and leukemias (96) have been approved by the FDA for therapeutic use (97, 98). One of the issues associated with CAR-T cells is that they can persist and be activated for long periods of time in the body, causing cytokine release syndrome (CRS). Symptoms of CRS manifest as fatigue, fever, nausea, cardiac failure, among other symptoms (99). CAR-NK therapies are being developed to overcome some of the issues associated with CAR-T cell therapy. Protocols have been developed to isolate NK cells from cord blood and expanded for use in patients. NK cells do not persist for long periods of time *in vivo* after infusion (100), do not cause GVHD, and can recognize tumor targets through intrinsic receptors (101). If SCID pigs can successfully develop human NK cells *de novo*, humanized SCID pig blood could be a source of NK cells. Six month old *ARTEMIS*^−/−^ Yorkshire SCID pigs are ~85 kg (personal observation), and thus according to IACUC guidelines, up to 1.2 L of blood could be collected for human NK cell isolation and used for CAR therapy research.

We envision several applications for a hu-PBL SCID pig in testing cell-based immunotherapies. As more CAR therapy targets are generated, it may be possible to test their efficacy and safety in a humanized SCID pigs that are xenografted with a human tumors. Other CAR therapies that are currently under development are CAR-T cells targeting CD20 (102), CD30 (103), CD33 (104), CD7 (105), and CAR-NK cells targeting CD33 (106) and CD19 (107). In addition, as the field of precision medicine continues to grow, a patient's tumor could be xenografted into a SCID pig and a therapy could be tested. Tumors in SCID pigs could be grown to a comparable size to those found in humans and would therefore be a more representative model compared to the limited size of tumors in mouse models. Similar, studies have been performed in hu-PBL-mice, in which interactions between human thyroid tumors and PBLs were studied (108).

### Improving targeting imaging techniques

Pigs are an excellent animal model for surgical and clinical imaging research. Due to their larger size, techniques that are used for humans in the clinics (PET, MRI, CT, US) can also be readily adapted for use in swine. There are immunocompetent pig models of cancer that exist with inducible mutations in p53 (15, 109, 110) and KRAS (111). Pigs with inducible tumors have previously been imaged with CT and MRI, which is proof of concept that these imaging techniques can be performed on pigs (110).

There are also practices that involve targeted imaging of tumors using small peptides and molecules. SCID mice have previously been used for such studies for ovarian (112), nasopharyngeal, breast (113), hepatic (114), lung cancer (115), and others. SCID mice are useful animal models for proof of concept studies that certain molecules and peptides can specifically bind to certain tumor types. After preliminary testing has been completed in mice, SCID pig models engrafted with human cells could then be used for testing these targeting techniques with respective imaging equipment that would be used in the clinics. As an example, human ovarian carcinomas expressing high levels of Claudin 3/4 expression will grow in SCID pigs (45). A *Clostridium perfringens enterotoxin* (CPE) peptide can specifically bind to Claudin 3/4 (112, 116), and such a SCID pig ovarian cancer model can be used as an imaging and therapeutic target of the CPE peptide in targeting ovarian carcinomas in such a way that it is translatable to human patients.

### Development of patient derived xenograft models in SCID pigs for personalized drug testing

Since SCID pigs have previously been shown to accept xenografts of human cancer cells (13), as well as pluripotent stem cells (24, 46), it would be expected that they would also accept solid tumor tissues as well. Patient derived xenograft (PDX) and cell derived xenograft models have previously been utilized in SCID mouse models for patient specific drug testing (117). SCID pig models can also be developed for these purposes. Due to higher similarity in metabolism between humans and pig (32) compared to mice, drug responses in the pig would likely lead to more directly comparable responses to those that would be found in humans (33). Additionally, the size of the pig would also allow representative drug doses to be tested that could be applied to future doses for human patients.

## Concluding remarks

Here we have described many of the novel uses of SCID pigs in oncology research involving the use of xenotransplantation of human tumor tissues, HSCs, and lymphoid tissues. The full potential of these animals will be realized when biocontainment facilities are more readily available and survivability of SCID pigs improved. Additionally, dissemination of handling protocols will be essential to prolonging the lives of these animals for long-term studies.

Research groups generating SCID pigs are at the forefront of creating a new animal model that can be used for translational preclinical research. We have learned an incredible amount of information by use of small animal mouse models for cancer research. However, in order for therapies to be developed and tested thoroughly, they now need to be evaluated in a larger animal model that better represents human disease states and which can provide realistic opportunities for improved modeling of imaging and surgical approaches. As such, we believe that SCID pig models will provide a foundation for researchers to gain valuable and translational results to improve patient outcomes in a clinical setting.

## Author contributions

AB wrote manuscript and designed figures. JC and CL edited and revised manuscript. CT wrote, edited, and revised manuscript.

### Conflict of interest statement

The authors declare that the research was conducted in the absence of any commercial or financial relationships that could be construed as a potential conflict of interest.

## Abbreviations

SCID: severe combined immunodeficiency
IL2Rγ: Interleukin 2 receptor gamma
RAG: recombination activating gene
HSCs: Hematopoietic stem cells
PBL: peripheral blood leukocytes
VDJ: variable, diversity and joining
TCR: T cell receptor
BCR: B cell receptor
DSB: double stranded break
NSG: NOD-SCID-IL2Rγ
IP: intraperitoneal
IV: intravenous
TREC: T cell receptor excision circles
GVHD: graft vs. host disease
BLT: bone marrow, liver and thymus
CAR: chimeric antigen receptor
CRS: cytokine release syndrome
PET: positron emission tomography
MRI: magnetic resonance imaging
CT: computer tomography
US: ultrasound
PDX: patient derived xenograft
CDX: cell derived xenograft.
