# Supplementary material for: Development of Severe Combined Immunodeficient (SCID) Pig Models for Translational Cancer Modeling: Future Insights on How Humanized SCID Pigs Can Improve Preclinical Cancer Research
Source: Front Oncol. 2018 Nov 30;8:559. doi: 10.3389/fonc.2018.00559 (PMC6284365; doi:10.3389/fonc.2018.00559)
Supplement: Supplementary file 1 [file Table_1.docx]

**Supplemental Tables**

| Supplemental Table 1. Ensemble accession numbers used for amino acid comparisons | | | |
| --- | --- | --- | --- |
| **Protein** | **Human** | **Pig** | **Mouse** |
| **SCF** | ENSG00000049130 | ENSSSCG00000035495 | ENSMUSG00000019966 |
| **TPO** | ENSG00000090534 | ENSSSCG00000023909 | ENSMUSG00000022847 |
| **Flt-3** | ENSG00000122025 | ENSSSCG00000009314 | ENSMUSG00000042817 |
| **IL-11** | ENSG00000095752 | ENSSSCG00000040725 | ENSMUSG00000004371 |
| **CXCL12** | ENSG00000107562 | ENSSSCG00000034973 | ENSMUSG00000061353 |
| **EPO** | ENSG00000130427 | ENSSSCG00000007673 | ENSMUSG00000029711 |
| **IL-15** | ENSG00000164136 | ENSSSCG00000009051 | ENSMUSG00000031712 |
| **IL-2** | ENSG00000109471 | ENSSSCG00000033267 | ENSMUSG00000027720 |
| **IL-4** | ENSG00000113520 | ENSSSCG00000014282 | ENSMUSG00000000869 |
| **IL-5** | ENSG00000113525 | ENSSSCG00000014278 | ENSMUSG00000036117 |
| **IL-6** | ENSG00000136244 | ENSSSCG00000020970 | ENSMUSG00000025746 |
| **IL-7** | ENSG00000104432 | ENSSSCG00000006161 | ENSMUSG00000040329 |
| **M-CSF** | ENSG00000184371 | ENSSSCG00000037449 | ENSMUSG00000014599 |
| **GM-CSF** | ENSG00000164400 | ENSSSCG00000023737 | ENSMUSG00000018916 |
| **G-CSF** | ENSG00000108342 | ENSSSCG00000017488 | ENSMUSG00000038067 |
